# Timing and outcomes of out-of-hospital traumatic cardiac arrest: results of a multicentre, retrospective cohort study

**DOI:** 10.1016/j.resplu.2026.101393

**Published:** 2026-06-26

**Authors:** Romana Erblich, Matthias Noitz, Marius Knöll, Helmut Trimmel, Wolfgang Voelckel, Luca Carenzo, Marius Rehn, Dominik Jenny, Jens Meier, Martin W. Dünser

**Affiliations:** a Department of Anaesthesiology and Critical Care Medicine, Kepler University Hospital and Johannes Kepler University, Linz, Austria; b OEAMTC Flugrettung, Vienna, Austria; c Karl Landsteiner Institute for Medical Simulation, Patient Safety and Emergency Medicine, Seebenstein, Austria; d Department of Anaesthesiology and Intensive Care Medicine, AUVA Trauma Centre Salzburg, Academic Teaching Hospital of the Paracelsus Medical University, Salzburg, Austria; e Department of Anaesthesia and Intensive Care Medicine, IRCCS Humanitas Research Hospital, Rozzano, Milano, Italy; f Air Ambulance Department, Division of Prehospital Services, Oslo University Hospital, Oslo, Norway; g Department of Research and Development, Norwegian Air Ambulance Foundation, Oslo, Norway; h Institute of Clinical Medicine, University of Oslo, Oslo, Norway

**Keywords:** Injury, Trauma, Out-of-hospital, On scene, Transport, Helicopter emergency medical service, Return of spontaneous circulation, Survival

## Abstract

**Background:**

Out-of-hospital traumatic cardiac arrest (TCA) is associated with high mortality. Outcomes may depend on the timing of TCA onset. This study aimed to characterize the timing of out-of-hospital TCA and its association with patient outcomes.

**Methods:**

In this multicentre, retrospective, observational study, patients with out-of-hospital TCA attended by a nationwide helicopter emergency medical service (HEMS) were included. TCA timing was stratified into three prehospital intervals: on scene before arrival of the first emergency medical service (EMS) unit, on scene after EMS arrival, and during HEMS transport. Primary outcomes were proportions of TCA across time intervals. Secondary outcomes included sustained return of spontaneous circulation (ROSC), 30-day survival, and 1-year survival in patients without criteria to withhold resuscitation. Multivariable regression was used to identify predictors of sustained ROSC and 1-year survival among patients sustaining TCA before EMS arrival.

**Results:**

Among 1532 patients, 90.2% sustained TCA before EMS arrival, 8.2% after EMS arrival, and 1.6% during HEMS transport. Sustained ROSC occurred in 20.8%, 47.2%, and 92.0% of patients, respectively (*p* < 0.001). One-year survival was 3.5%, 10.0%, and 15.0% (*p* < 0.001), respectively. In patients with TCA before EMS arrival, an initial electrocardiographic rhythm other than asystole was the only independent predictor of sustained ROSC and 1-year survival.

**Conclusion:**

Nine out of ten out-of-hospital TCAs occur before EMS arrival. Timing of out-of-hospital TCA is associated with resuscitation success and survival. Meaningful survival rates are, however, achievable irrespective of TCA onset. This supports resuscitation efforts in all patients with out-of-hospital TCA and no criteria to withhold resuscitation.

## Highlights

• Nine out of ten out-of-hospital traumatic cardiac arrests occur on scene before arrival of the first EMS unit.
• Timing of out-of-hospital traumatic cardiac arrest is associated with the odds of sustained ROSC, short- and long-term survival.
• Sustained ROSC was achievable also in patients with traumatic cardiac arrest occurring on scene before EMS arrival.
• Short- and long-term survival was highest when traumatic cardiac arrest occurred after EMS arrival or during HEMS transport.
• A non-asystolic initial ECG rhythm predicted ROSC and 1-year survival in those with traumatic cardiac arrest on scene before EMS arrival.

## Introduction

Traumatic cardiac arrest (TCA) predominantly affects young, previously healthy subjects.^1^, ^2^ Nonetheless TCA is associated with excessively high mortality rates.^3^, ^4^ This even led some physicians to consider resuscitation efforts in patients with prehospital TCA as futile.^5^, ^6^ One study reported that resuscitation was attempted in less than 30% of out-of-hospital TCA cases.^7^ More recent data from civilian settings, however, highlighted that TCA is a survivable condition, and resuscitation efforts are warranted.^8^, ^9^, ^10^ Most TCAs occur during the prehospital phase, leaving only an extremely brief time window for effective interventions.^11^ On the other hand, our current understanding of TCA outcomes is selectively biased toward hospital-level events, often excluding prehospital deaths.^12^, ^13^, ^14^, ^15^ This may overestimate survival rates, particularly of patients, who sustain TCA prior to hospital admission. Therefore, gaining a more detailed understanding of the timing and timing-dependent outcomes of out-of-hospital TCA is crucial, as this knowledge is likely to directly impact the feasibility, type and potential survival benefits of prehospital therapies to reverse TCA.

In this study, we sought to determine the timing of out-of-hospital TCA (on scene before EMS arrival, on scene after EMS arrival, during transport) and its association with short- and long-term outcomes. In addition, predictors of a sustained return of spontaneous circulation (ROSC) and 1-year survival in patients sustaining TCA on scene prior to EMS arrival were evaluated. Given that both epidemiology and aetiology of out-of-hospital TCA differ between military and civilian settings, it is important to emphasize that this study was conducted in a civilian setting.

## Patients and methods

### Study design

This analysis was designed as a multicentre, retrospective, observational cohort study. It included data of patients treated by the teams of thirteen helicopter emergency medical services (HEMS) of the OEAMTC Flugrettung [Österreichischer Automobil-, Motorrad- und Touring Club (OEAMTC), Vienna, Austria] from January 1, 2010 until December 31, 2019. The present study was part of a larger research project aiming to investigate the characteristics, treatment, as well as short- and long-term outcomes of patients with out-of-hospital cardiac arrest managed by HEMS teams of the OEAMTC Flugrettung. The research project was approved by the Ethics Committee of the Federal State of Lower Austria (GS4-EK-4/703-2020). Due to the retrospective design of the study written informed consent was waived. The results of previous analyses focusing on the outcomes of selected patient groups with out-of-hospital cardiac arrest are available elsewhere.^16^, ^17^, ^18^ The Strengthening the Reporting of Observational Studies in Epidemiology (STROBE) guidelines informed the structure and content of this manuscript (Electronic Supplementary Material Table 1).^19^

### Study setting

The OEAMTC Flugrettung is the only organization contractually bound to provide nationwide HEMS in Austria. During the observation period, the OEAMTC Flugrettung operated sixteen HEMS bases throughout the country. Medical data of thirteen bases were included into the electronic database. The HEMS teams consisted of a prehospital emergency physician (75% anaesthesiologists), a flight paramedic, and a pilot. Throughout Austria, physician staffed EMS units (either rapid response cars or HEMS) are dispatched to life-threatening trauma- and non-trauma-related emergencies by regional ambulance control centers. The decision whether a physician-staffed rapid response car or a HEMS team was dispatched to a patient with TCA was based on availability, accessibility, and the estimated time needed to reach the scene. During the study period, no HEMS unit carried blood, and no HEMS-wide TCA resuscitation protocol existed. A prior analysis detailed the rate and effects of resuscitation interventions in 996 TCA patients managed by this nationwide HEMS system.^18^ Similarly, no standard operating procedure regarding indications for endotracheal intubation in major trauma patients had been implemented, and the process of rapid sequence induction in critically ill trauma patients was not standardized throughout the national service.

### Inclusion and exclusion criteria

The electronic database of the OEAMTC Flugrettung was screened for primary missions responding to patients with a confirmed out-of-hospital cardiac arrest. All patients irrespective of age, who sustained TCA in the out-of-hospital setting, were eligible for study inclusion. Patients with out-of-hospital cardiac arrest from non-traumatic aetiology and subjects, in whom the time interval of TCA onset could not be determined as well as those with cardiac arrest due to burn injuries were excluded.

### Study variables

The following variables were extracted from the electronic database: age, sex, type of accident (including whether trauma occurred in an alpine setting), mechanism of injury (blunt vs. penetrating), injury pattern (as determined by the HEMS physician using clinical acumen with or without the use of point-of-care sonography), bystander witness status of the onset of TCA, bystander cardiopulmonary resuscitation (including bystander defibrillation), timing of TCA, HEMS response time (duration from HEMS dispatch alarm to arrival on scene), inability to immediately access the patient at the scene of accident (e.g. due to patient entrapment in a car wreck, patient located in alpine terrain requiring aerial rescue, burial, hanging), presence of ERC suggested criteria to withhold resuscitation, initiation of resuscitation or pronouncement of death without initiating resuscitation (including reasons), initial electrocardiographic rhythm, and the immediate outcome of resuscitation (sustained ROSC vs. no sustained ROSC). The survival status at thirty days and one year after TCA of study patients with Austrian nationality, in whom sustained ROSC had been achieved, was retrieved from the Austrian registry of deaths (Statistik Austria; Vienna, Austria). The survival status of study patients with sustained ROSC but other nationalities could not be retrieved.

### Definitions

In accordance with the international literature^1^ and guidelines,^20^ TCA was defined as the clinical phenotype of an unresponsive patient without a palpable central pulse supposedly due to a traumatic cause. Other external causes of cardiac arrest (e.g. drowning, electrocution, accidental hypothermia) were not considered as TCA. Similarly, trauma patients, in whom mission protocols implied that cardiac arrest resulted from a medical cause, were not classified as having sustained a TCA. The timing of TCA was stratified into the following three prehospital intervals: (1) on scene before arrival of the first EMS unit, (2) on scene after arrival of the first EMS unit, and (3) during HEMS transport from the scene to the hospital. In case of repeated TCAs, patients were stratified based on the timing of the first TCA. As informed by the European Resuscitation Council (ERC) guidelines,^20^ the following conditions were considered criteria to withhold resuscitation: signs of irreversible death, massive trauma incompatible with survival (e.g. decapitation, extensive cardiac destruction, massive head injury with loss of brain tissue), and no signs of life within the preceding 15 min. Sustained ROSC was defined in line with the Utstein criteria as the restoration of a palpable central pulse together with an autonomous electrocardiographic rhythm until admission and transfer of the patient to medical staff at the receiving hospital or for at least 20 min after the cessation of cardiopulmonary resuscitation.^21^

### Study objectives

The primary objective of this study was to determine the proportion of patients sustaining TCA during the three prehospital time intervals. Secondary objectives were the rates and odds of sustained ROSC, 30-day, and 1-year survival in patients with TCA stratified into the three prehospital time intervals. In addition, we sought to identify predictors of sustained ROSC and 1-year survival in patients, who developed TCA on scene before arrival of the first EMS unit. Secondary objectives were only determined in patients without ERC-suggested criteria to withhold resuscitation.

### Data processing

Only relevant sections of the medical records of the electronic database were transmitted by the data management team of the OEAMTC Flugrettung strictly complying with pseudo-anonymization and data minimization requirements. Following data extraction, quality control checks were performed to identify syntax or entry errors. Wherever possible, these errors were rectified. No data imputation methods were used to compensate for missing values.

### Statistical analysis

All statistical analyses were performed with the SPSS software package (SPSS 30.0.0; IBM, Armonk, New York). Descriptive methods were used to report the characteristics of the study population. Following evaluation of normality distribution using Shapiro-Wilks tests, continuous variables were presented as median values with interquartile ranges (IQR). Absolute numbers with percentages and their 95% confidence intervals (95%CI) were used to report categorical variables. The rates of sustained ROSC, 30-day and 1-year survival were compared between the three prehospital time intervals using Chi^2^-tests. Crude odds ratios and their 95%CIs for the same outcome variables were calculated for each prehospital time interval using binary logistic regression models. Given the exploratory nature of the secondary study endpoints, no adjustments were made for multiple testing and *p*-values < 0.05 considered to indicate statistical significance. To identify predictors of sustained ROSC and 1-year survival in patients with TCA on scene before arrival of the first EMS unit, the following variables were compared between patients with and without sustained ROSC or 1-year survival: sex, age [stratified into pediatric (<18 years), adult (18–64 years) and geriatric (>64 years) age groups], trauma occurring in an alpine setting, mechanism of trauma, injury pattern, bystander witness status of TCA onset, bystander cardiopulmonary resuscitation, HEMS response time, ability of EMS to immediately access the patient at the scene of accident, and the initial electrocardiographic rhythm. Variables that were not strongly correlated with each other (defined as a Spearman correlation coefficient ≤0.5) and with significant (*p* < 0.05) between-group differences (evaluated with the use of Chi^2^-tests for categorical variables and the Mann-Whitney-*U* test for HEMS response time), were then included into a multivariable logistic regression model using sustained ROSC (model I) or 1-year survival (model II) as the dependent variable. Adjusted odds ratios with their 95%CIs were calculated to quantify the strength, direction, and statistical significance of the independent association of each variable with the outcome.

## Results

**Fig. 1:**
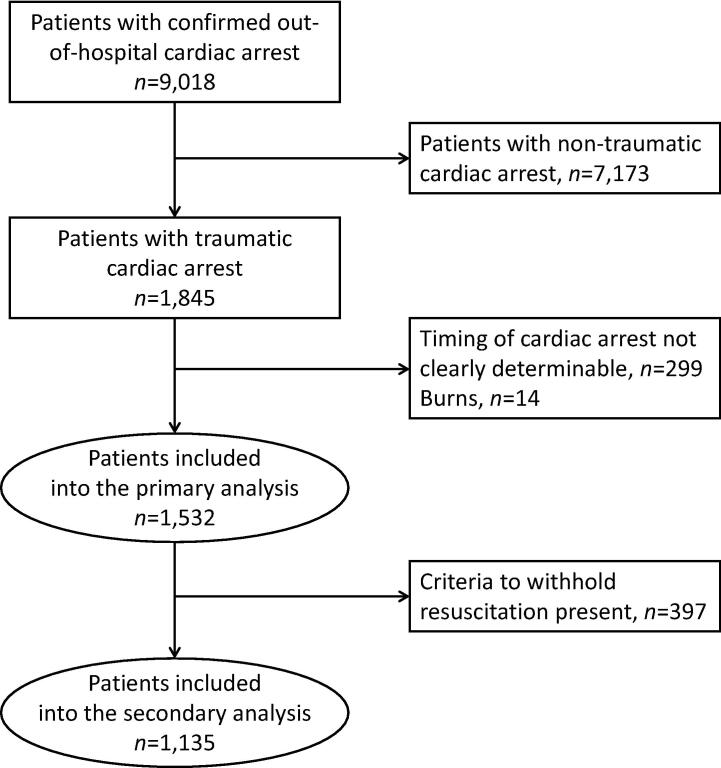
**Study flow diagram**.

Of 9018 patients with confirmed out-of-hospital cardiac arrest, 1845 patients were classified as TCA. After application of exclusion criteria, a total of 1532 subjects were included into the statistical analysis investigating the primary study endpoint (Fig. 1). Characteristics of the study population are summarized in Table 1. Resuscitation was initiated in 817/1532 (53.3%) patients with TCA. Of the remaining 715 patients (all sustaining TCA before arrival of the first EMS unit), in whom resuscitation was not initiated and who were pronounced dead on scene, ERC-suggested criteria to withhold resuscitation were clearly documented in 397 (55.5%) subjects. In the remaining 318 patients, the following reasons for not initiating resuscitation were identified: massive injuries other than trauma incompatible with life (*n* = 61), prolonged strangulation without clear time interval given (*n* = 60), gunshot injury to the head (*n* = 30), dilated pupils unreactive to light (*n* = 29), patient not accessible by EMS/HEMS teams (*n* = 28), triage situation (*n* = 1), and unknown reason (*n* = 109). The median time from HEMS activation to arrival on scene was 13 (IQR, 10–17) min and did not differ between patients stratified into the three prehospital time intervals (*p* = 0.40). HEMS arrival on scene occurred within 20 min after activation in 82.8% of study patients (Electronic Supplementary Material Fig. 1). The median time HEMS spent on scene was 26 (IQR, 17–39) min. HEMS transport to hospital of patients, in whom ROSC could be achieved, took a median time of 10 (IQR, 7–15) min.

**Table 1:** Characteristics of study patients and traumatic cardiac arrest scenarios stratified by the three prehospital time intervals.

		**TCA on scene before arrival of first EMS unit**	**TCA on scene after arrival of first EMS unit**	**TCA during HEMS transport to hospital**
*n*		1382	125	25
Male sex	*n* (%)	1122 (81.4)	103 (82.4)	23 (92.0)
Age	years	52 (36–67)	53 (30–64)	49 (27–71)
Age categories	*n* (%)			
Pediatric (<18 years)		63 (4.6)	9 (7.2)	1 (4.0)
Adult (18–64 years)		924 (66.9)	87 (69.6)	17 (68.0)
Geriatric (>64 years)		387 (28.0)	29 (23.2)	7 (28.0)
Missing		8 (0.6)	0 (0)	0 (0)
**Type of accident**				
Road		465 (33.6)	56 (44.8)	12 (48.0)
Work		201 (14.5)	20 (16.0)	6 (24.0)
Household		250 (18.1)	24 (19.2)	4 (16.0)
Sports/outdoor		313 (22.6)	17 (13.6)	3 (12.0)
Other/missing		153 (11.1)	8 (6.4)	0 (0)
Trauma occurring in alpine setting	*n* (%)	264 (19.1)	8 (6.4)	1 (4.0)
Mechanism of trauma	*n* (%)			
Blunt		1274 (92.2)	117 (93.6)	25 (100.0)
Penetrating		100 (7.2)	7 (5.6)	0 (0)
Missing		8 (0.6)	1 (0.8)	0 (0)
Injury pattern	*n* (%)			
Multiple trauma		845 (61.1)	83 (66.4)	19 (76.0)
Severe traumatic brain injury		256 (18.5)	24 (19.2)	5 (20.0)
Severe chest injury		95 (6.9)	16 (12.8)	1 (4.0)
Asphyxia/hanging		163 (11.8)	0 (0)	0 (0)
Missing		23 (1.7)	2 (1.6)	0 (0)
Bystander witnessed onset of TCA	*n* (%)		Not applicable	Not applicable
Witnessed		262 (19.0)		
Unwitnessed		832 (60.2)		
Missing		288 (20.8)		
Bystander cardiopulmonary resuscitation	*n* (%)	495 (35.8)	Not applicable	Not applicable
Bystander defibrillation	*n* (%)	1 (0.1)	Not applicable	Not applicable
Patient not immediately accessible	*n* (%)	509 (36.8)	28 (22.4)	4 (16.0)
Resuscitation initiated	*n (%)*	667 (48.3)	125 (100)	25 (100)
Criteria to withhold resuscitation present	*n* (%)	397 (28.7)	Not applicable	Not applicable
Irreversible signs of death		175 (12.7)		
Massive injuries incompatible with life		162 (11.7)		
No signs of life within the preceding 15 min		60 (4.3)		
Initial electrocardiographic rhythm	*n* (%)			
Asystole		430 (31.1)	21 (16.8)	5 (20.0)
Pulseless electrical activity		109 (7.9)	48 (38.4)	9 (36.0)
Shockable rhythm		13 (0.9)	7 (5.6)	4 (16.0)
Missing		830 (60.1)	49 (39.2)	7 (28.0)

HEMS, helicopter emergency medical service; TCA, traumatic cardiac arrest.

Data are given as absolute values with percentages or median values with interquartile ranges.

### Primary study objective: timing of out-of-hospital traumatic cardiac arrest

**Fig. 2:**
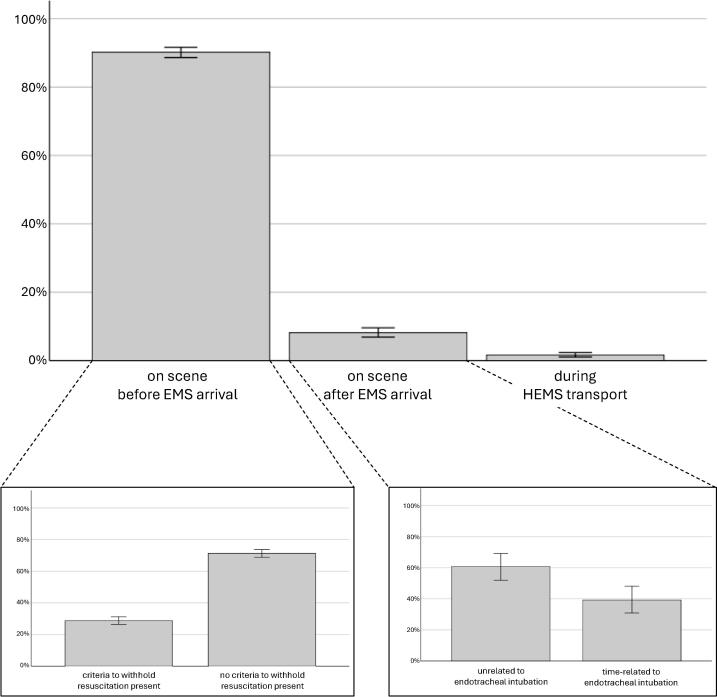
**Proportion of patients experiencing out-of-hospital traumatic cardiac arrest in each of the three prehospital time intervals**. EMS, emergency medical services.

TCA occurred on scene before arrival of the first EMS unit in 1382 patients corresponding to 90.2% (95%CI, 88.6–91.7%) of all 1532 study patients. ERC-suggested criteria to withhold resuscitation were present in 397 of these 1382 [28.7% (95%CI, 26.4–31.2%)] subjects. Hundred-twenty-five patients [8.2% (95%CI, 6.8–9.6%)] sustained TCA on scene after arrival of the first EMS unit. In 25 [1.6% (95%CI, 1.1–2.4%)] study patients, TCA occurred during HEMS transport from the scene to the hospital. A post-hoc analysis of the 125 patients, who sustained TCA on scene after arrival of the first EMS unit, revealed that 49 subjects [39.2% (95%CI, 30.6–48.3%)] sustained TCAs in the context (during or within <5 min) of endotracheal intubation (Fig. 2).

### Secondary study objective: outcomes of TCA patients stratified by the timing of out-of-hospital traumatic cardiac arrest

One-thousand-one-hundred-thirty-five patients were included into the statistical analysis investigating the secondary study endpoints (Fig. 1). The rates of sustained ROSC, 30-day and 1-year survival could be retrieved in 1135 (100%), 1074 (94.6%), and 1074 (94.6%) of these patients. Resuscitation was initiated in 817/1135 (72.0%) patients [667/985 (67.7%) patients with TCA on scene before EMS arrival; 125/125 (100%) patients with TCA on scene after EMS arrival; 25/25 (100%) patients with TCA during HEMS transport].

**Fig. 3:**
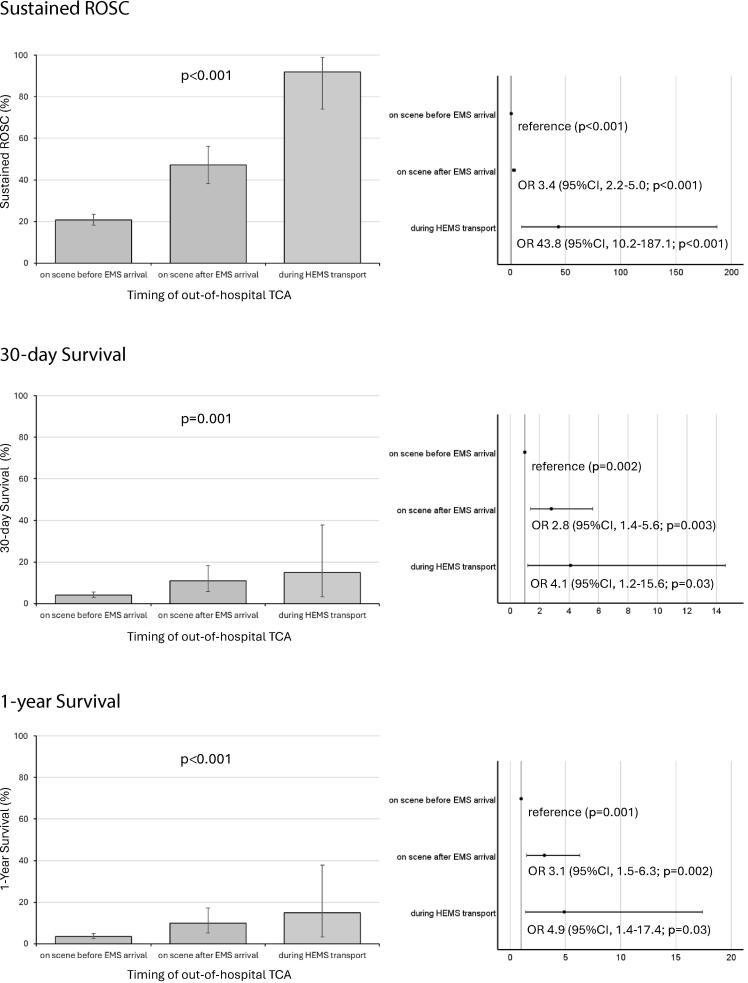
**Rates and odds of sustained ROSC, 30-day and 1-year survival in patients without contraindications to resuscitation (stratified into the three prehospital time intervals when traumatic cardiac arrest occurred)**. CI, confidence interval; EMS, emergency medical services; HEMS, helicopter emergency medical services; OR, odds ratio; ROSC, return of spontaneous circulation; TCA, traumatic cardiac arrest.

The rates and odds of sustained ROSC, 30-day and 1-year survival are displayed in Fig. 3. The rates of sustained ROSC significantly differed between the three prehospital time intervals: 20.8% (95%CI, 18.3–23.5%) (205/985) among study patients, in whom TCA occurred on scene before arrival of the first EMS unit, 47.2% (95%CI, 38.2–56.3%) (59/125) on scene after arrival of the first EMS unit, and 92.0% (95%CI, 74.0–99.0%) (23/25) during HEMS transport from the scene to the hospital. The rates of 30-day survival significantly differed between the three prehospital time intervals: 4.1% (95%CI, 3.0–5.6%) (39/944) among study patients, in whom TCA occurred on scene before arrival of the first EMS unit, 10.9% (95%CI, 5.8–18.3%) (12/110) on scene after arrival of the first EMS unit, and 15.0% (95%CI, 3.2–37.9%) (3/20) during HEMS transport from the scene to the hospital. Similarly, the rates of 1-year survival differed between the three prehospital time intervals: 3.5% (95%CI, 2.4–4.9%) (33/944) among study patients, in whom TCA occurred on scene before arrival of the first EMS unit, 10.0% (95%CI, 5.1–17.2%) (11/110) on scene after arrival of the first EMS unit, and 15.0% (95%CI, 3.2–37.9%) (3/20) during HEMS transport from the scene to the hospital. Sustained ROSC [59.2% (29/49) vs. 39.5% (30/76), *p* = 0.03], 30-day [23.1% (9/39) vs. 4.2% (3/71), *p* = 0.002] and 1-year [20.5% (8/39) vs. 4.2% (3/71), *p* = 0.006] survival rates were significantly higher among patients, who sustained TCA on scene after arrival of the first EMS unit, when cardiac arrest occurred in the context of endotracheal intubation compared with those, in whom TCA was unrelated to endotracheal intubation.

### Secondary study objective: predictors of sustained ROSC and 1-year survival in patients with traumatic cardiac arrest on scene before arrival of the first EMS unit

Nine-hundred-eighty-five patients with TCA on scene before arrival of the first EMS unit had no contraindication to resuscitation and were included into the analysis to identify predictors of sustained ROSC and 1-year survival. Significant group differences between subjects with or without sustained ROSC as well as between survivors and non-survivors at one year following TCA are shown in Electronic Supplementary Material Tables 2 and 3. In both multivariate models, only the initial electrocardiographic rhythm was independently associated with sustained ROSC or 1-year survival. An initial electrocardiographic rhythm other than asystole was independently associated with sustained ROSC and 1-year survival (Table 2).

**Table 2:** Multivariate regression models to predict sustained ROSC (Model I) and one-year survival (Model II) in patients experiencing traumatic cardiac arrest on scene before EMS arrival.

**Model I – Dependent variable: sustained ROSC**				
	**Variable type**	**Odds ratio**	**95% CI**	***p*-value**
Penetrating mechanism of trauma	Categorical	0.52	0.10–2.64	0.43
Injury pattern	Categorical			
Multiple trauma		Reference		0.75
Severe traumatic brain injury		1.12	0.58–2.15	0.74
Severe chest injury		0.72	0.34–1.54	0.40
Asphyxia/hanging		0.58	0.07–4.89	0.62
Bystander witnessed onset of TCA	Categorical	1.44	0.81–2.57	0.21
Bystander CPR	Categorical	1.27	0.72–2.25	0.41
Patient not immediately accessible	Categorical	0.98	0.52–1.86	0.96
Initial electrocardiographic rhythm	Categorical			
Asystole		Reference		<0.001
Pulseless electrical activity		4.17	2.39–7.27	<0.001
Shockable		3.19	0.80–12.76	0.10
**Model II – Dependent variable: one-year survival**				
Bystander witnessed onset of TCA	Categorical	1.69	0.44–6.42	0.45
Bystander CPR	Categorical	3.84	0.46–31.75	0.21
Initial electrocardiographic rhythm	Categorical			
Asystole		Reference		0.05
Pulseless electrical activity		0.97	0.19–5.03	0.97
Shockable		8.85	1.45–53.90	0.02

CI, confidence interval; CPR, cardiopulmonary resuscitation; EMS, emergency medical services; ROSC, return of spontaneous circulation; TCA, traumatic cardiac arrest.

## Discussion

In this multicentre, retrospective cohort study, we analyzed 1532 patients with out-of-hospital TCA attended by teams of a nationwide HEMS system in Austria. Based on the timing of TCA onset, patients were stratified into three prehospital time intervals. Since the exact times of neither the moment of injury nor the onset of TCA were known, we categorized study patients into three pragmatic time intervals (on scene before arrival of the first EMS unit, on scene after arrival of the first EMS unit, during HEMS transport to the hospital). Although these prehospital time intervals are universally applicable to prehospital trauma care, the duration of each phase might still be context- (e.g. TCA occurring in alpine settings) and system-dependent. It is, therefore, important to remember that only patients with TCA, who were attended by HEMS teams, were included into this cohort. This might have introduced a selection bias, as the epidemiology and aetiologies of TCA cases attended by other physician-staffed EMS units (e.g. physician-staffed rapid response cars) could be different both in timing and outcomes.

Nine out of ten patients in our study sustained TCA on scene before arrival of the first EMS unit. Given that >80% of study patients were reached by the HEMS team within 20 min of activation and other EMS units typically arrive first, most TCAs occurring on scene before EMS arrival have likely occurred within 15–20 min following injury or even earlier. This supports recent data highlighting the rapid onset of TCA in the majority of patients and underscores the extremely short time window for therapeutic interventions in these subjects.^11^ Our findings are in line with the results of three large database analyses reporting that 80%,^22^ 89.9%^23^ and 91.6%^3^ of trauma patients were already in TCA at arrival of the advanced trauma team or EMS, respectively. Even though we could not determine the underlying cause of TCA, criteria to withhold resuscitation as suggested by contemporary guidelines were clearly documented in 28.7% of patients sustaining TCA before EMS arrival. Resuscitation was not initiated in another 20% of study patients for other reasons such as massive trauma, prolonged strangulation, gunshot injuries to the head, or inaccessibility to EMS teams. This suggests that injury severity or circumstances were likely incompatible with survival in at least one third of TCA patients in our cohort. Similarly, the nature of injuries and delays until HEMS arrival could explain why study patients developing TCA on scene before EMS arrival had the lowest rates of sustained ROSC as well as short- and long-term survival. Despite these facts, sustained ROSC could be achieved in 20.8% (95%CI, 18.3–23.5%) of patients sustaining TCA on scene before EMS arrival, and 3.5% (95%CI, 2.4–4.9%) survived to one year. Accordingly, resuscitation efforts are warranted also in this group of TCA patients. As suggested by recent studies,^22^, ^24^ the multivariable analyses of our study highlight that the initial electrocardiographic rhythm could be a valuable indicator if sustained ROSC and long-term survival can be achieved in these patients or not. Taking these and prior research^22^, ^24^ into account, asystole as the initial electrocardiographic rhythm is an ominous sign and might make emergency physicians reconsider the meaningfulness of prolonged resuscitation efforts or use of advanced interventions.

Only one in ten patients developed out-of-hospital TCA in the attendance of EMS or during HEMS transport to the hospital in this cohort. This is a striking observation and might be a signal that TCA can effectively be prevented by interventions delivered by EMS including HEMS after arrival on scene. It could also reflect a biological selection bias of patients with the largest physiologic reserve to survive until EMS or HEMS arrival. Prior reports found that occurrence of TCA in the attendance of EMS was a positive predictor of survival.^12^, ^22^, ^25^, ^26^ The results of our study confirm these findings. The significant differences in the rates of sustained ROSC and survival between the three prehospital time intervals should, moreover, inform the design of future research projects on TCA, when reporting outcomes from out-of-hospital TCA. Out of our cohort, one group of patients experiencing TCA on scene after EMS arrival appears particularly noteworthy. These were patients whose TCA occurred in temporal relationship with prehospital endotracheal intubation. Although no causality between the two events can be established based on these results, it is an important finding of our analysis. Earlier studies have emphasized the risks associated with prehospital rapid sequence induction and endotracheal intubation in major trauma patients,^27^, ^28^ particularly those at high risk of bleeding.^29^, ^30^, ^31^

Important limitations need to be considered when interpreting the results of our study. First, this was a retrospective data analysis of a large HEMS database including a 10-years observation period. Accordingly, our study is subject to inherent limitations, such as incomplete or missing data, potential data misclassifications, and time-related confounders. Although considered unlikely, retrospective misclassification of the true onset of TCA based on mission reports, affecting the validity of the primary study endpoint might have occurred in some patients. Furthermore, missing data were common, particularly for selected variables such as the initial electrocardiographic rhythm informing secondary endpoints of this study. Second, we could only evaluate survival as a binary variable. Reporting functional outcomes following TCA would have been a more-patient centered endpoint.^32^ Third, we only evaluated prehospital factors associated with the odds of sustained ROSC and survival. This does not take into account other factors known to affect patient outcomes after major trauma (e.g. type and level of hospital the patient was admitted to, in-hospital care). Fourth, although a total of 1532 patients were included into the statistical analysis, the number of subjects in some of the study groups were low and only allowed to report estimates with rather wide confidence intervals. Finally, it is an important limitation of our study that we could not determine the causes of out-of-hospital TCA, as this might have allowed for better understanding of both the reversibility of TCA and potential therapeutic interventions in these patients.

In conclusion, nine out of ten out-of-hospital TCAs occur before EMS arrival. Timing of out-of-hospital TCA is associated with resuscitation success and survival. Meaningful survival rates are, however, achievable irrespective of TCA onset. This supports resuscitation efforts in all patients with out-of-hospital TCA and no criteria to withhold resuscitation.

## CRediT authorship contribution statement

**Romana Erblich:** Writing – original draft, Visualization, Validation, Methodology, Investigation, Data curation, Conceptualization. **Matthias Noitz:** Writing – review & editing, Methodology, Investigation. **Marius Knöll:** Writing – review & editing, Investigation. **Helmut Trimmel:** Writing – review & editing, Investigation, Data curation. **Wolfgang Voelckel:** Writing – review & editing, Investigation, Data curation. **Luca Carenzo:** Writing – review & editing, Investigation. **Marius Rehn:** Writing – review & editing, Investigation. **Dominik Jenny:** Writing – review & editing, Investigation. **Jens Meier:** Writing – review & editing, Supervision, Methodology, Formal analysis. **Martin W. Dünser:** Writing – original draft, Visualization, Validation, Supervision, Methodology, Investigation, Formal analysis, Conceptualization.

## Ethics approval and consent to participate

This analysis was part of a larger research project aiming to investigate the characteristics, treatment, as well as short- and long-term outcomes of patients with out-of-hospital cardiac arrest managed by teams of the OEAMTC Flugrettung. The research project was evaluated and approved by the Ethics Committee of the Federal State of Lower Austria (GS4-EK-4/703-2020). Due to the retrospective design of the study written informed consent was waived.

## Consent for publication

Not applicable.

## Availability of data and materials

The datasets used and/or analyzed during the current study are available from the corresponding author on reasonable request.

## Funding

This research was funded by institutional funds only.

## Declaration of competing interest

None of the authors has a financial or non-financial conflict of interest regarding drugs, techniques or methods discussed in this manuscript.

## Appendix A Supplementary material

Supplementary Data 1 STROBE checklist and additional results.
